# Testing the benefits of conservation set‐asides for improved habitat connectivity in tropical agricultural landscapes

**DOI:** 10.1111/1365-2664.13472

**Published:** 2019-08-19

**Authors:** Sarah A. Scriven, Kimberly M. Carlson, Jenny A. Hodgson, Colin J. McClean, Robert Heilmayr, Jennifer M. Lucey, Jane K. Hill

**Affiliations:** ^1^ Department of Biology University of York York UK; ^2^ Department of Natural Resources and Environmental Management University of Hawai'i Mānoa Honolulu HI USA; ^3^ Institute of Integrative Biology University of Liverpool Liverpool UK; ^4^ Department of Environment and Geography University of York York UK; ^5^ Environmental Studies Program and Bren School of Environmental Science & Management University of California Santa Barbara CA USA; ^6^ Department of Zoology University of Oxford Oxford UK

**Keywords:** agriculture, Borneo, climate change, fragmentation, High Conservation Value, Incidence Function Model, landscape colonization, sustainable palm oil

## Abstract

1. Habitat connectivity is important for tropical biodiversity conservation. Expansion of commodity crops, such as oil palm, fragments natural habitat areas, and strategies are needed to improve habitat connectivity in agricultural landscapes. The Roundtable on Sustainable Palm Oil (RSPO) voluntary certification system requires that growers identify and conserve forest patches identified as High Conservation Value Areas (HCVAs) before oil palm plantations can be certified as sustainable. We assessed the potential benefits of these conservation set‐asides for forest connectivity.
2. We mapped HCVAs and quantified their forest cover in 2015. To assess their contribution to forest connectivity, we modelled range expansion of forest‐dependent populations with five dispersal abilities spanning those representative of poor dispersers (e.g. flightless insects) to more mobile species (e.g. large birds or bats) across 70 plantation landscapes in Borneo.
3. Because only 21% of HCVA area was forested in 2015, these conservation set‐asides currently provide few connectivity benefits. Compared to a scenario where HCVAs contain no forest (i.e. a no‐RSPO scenario), current HCVAs improved connectivity by ~3% across all dispersal abilities. However, if HCVAs were fully reforested, then overall landscape connectivity could improve by ~16%. Reforestation of HCVAs had the greatest benefit for poor to intermediate dispersers (0.5–3 km per generation), generating landscapes that were up to 2.7 times better connected than landscapes without HCVAs. By contrast, connectivity benefits of HCVAs were low for highly mobile populations under current and reforestation scenarios, because range expansion of these populations was generally successful regardless of the amount of forest cover.
4. *Synthesis and applications*. The Roundtable on Sustainable Palm Oil (RSPO) requires that High Conservation Value Areas (HCVAs) be set aside to conserve biodiversity, but HCVAs currently provide few connectivity benefits because they contain relatively little forest. However, reforested HCVAs have the potential to improve landscape connectivity for some forest species (e.g. winged insects), and we recommend active management by plantation companies to improve forest quality of degraded HCVAs (e.g. by enrichment planting). Future revisions to the RSPO's Principles and Criteria should also ensure that large (i.e. with a core area >2 km^2^) HCVAs are reconnected to continuous tracts of forest to maximize their connectivity benefits.

## Abstract 2

The Roundtable on Sustainable Palm Oil (RSPO) requires that High Conservation Value Areas (HCVAs) be set aside to conserve biodiversity, but HCVAs currently provide few connectivity benefits because they contain relatively little forest. However, reforested HCVAs have the potential to improve landscape connectivity for some forest species (e.g. winged insects), and we recommend active management by plantation companies to improve forest quality of degraded HCVAs (e.g. by enrichment planting). Future revisions to the RSPO's Principles and Criteria should also ensure that large (i.e. with a core area >2 km^2^) HCVAs are reconnected to continuous tracts of forest to maximize their connectivity benefits.

## 1 INTRODUCTION

Agricultural expansion has reduced the extent of natural habitats globally, and more than 12% of the Earth's ice‐free land surface is now under crop production (Ramankutty, Evan, Monfreda, & Foley, 2008). With demand for cropland expected to increase (Laurance, Sayer, & Cassman, 2014), decisions about how to conserve biodiversity within agricultural landscapes are of critical importance. Conservation of biodiversity in fragmented landscapes requires that habitat networks connect remaining areas of natural habitat to facilitate range shifts under climate change (Saura, Bodin, & Fortin, 2014) and maintain meta‐population dynamics (Hanski, 1994). Thus, there is an urgent need to determine how existing habitat networks facilitate movement of species across patchy landscapes (Hodgson et al., 2011).

Loss of habitat connectivity is of great concern in the tropics, where rapid expansion of commodity agriculture has resulted in widespread loss and fragmentation of forest (Hosonuma et al., 2012). In many areas, formerly extensive and contiguous forests now persist as isolated remnants scattered across vast agricultural matrices (Hill et al., 2011), and this conversion of forest to agriculture is accompanied by biodiversity losses (Laurance et al., 2014). Agricultural lands may also impede the dispersal of forest‐dependent species (Scriven, Beale, Benedick, & Hill, 2017), and hence their ability to track climate change. Land‐use and land‐cover changes are likely to interact with climate change to exacerbate the effects of fragmentation in tropical ecosystems by reducing suitable habitat availability (e.g. Nowakowski et al., 2017; Senior, Hill, González del Pliego, Goode, & Edwards, 2017). When current species distributions do not overlap with the locations of future suitable habitats under climate change (e.g. see Colwell, Brehm, Cardelús, Gilman, & Longino, 2008), populations are likely to decline in landscapes with poor connectivity (Newmark, Jenkins, Pimm, Mcneally, & Halley, 2017). Therefore, effective conservation measures that preserve forest connectivity are needed to support species persistence.

In Southeast Asia, the oil palm, pulp and paper, rubber and logging industries have driven lowland rainforest clearance (Carlson et al., 2018; Gaveau et al., 2016). As a result, few lowland forests outside of public protected areas remain (Curran et al., 2004). Given the projected growth in palm oil demand (Carrasco, Larrosa, & Edwards, 2014) and governments' interests in the palm oil industry as a vehicle for economic growth (Sayer, Ghazoul, Nelson, & Boedhihartono, 2012), as well as the substantial negative effects of oil palm agriculture on biodiversity (Meijaard et al., 2018), strategies are needed to reduce biodiversity losses in oil palm landscapes (Lucey et al., 2017). Conservation set‐asides are one approach used to meet such conservation goals (Green, Cornell, Scharlemann, & Balmford, 2005). To encourage such set‐asides, voluntary sustainability certification standards such as the Roundtable on Sustainable Palm Oil (RSPO) require members to identify and conserve areas within plantations that support High Conservation Values (HCVs; Senior, Brown, Villalpando, & Hill, 2015). HCVs are biological, social or cultural values of critical importance that are split into six broad types. Types 1–4 are important environmental values (e.g. for species diversity and ecosystem services), whilst types 5–6 are important for the livelihoods of local communities (e.g. community needs and cultural values) (see Senior et al., 2015, for a full description of HCV types). In the humid tropics, HCV types 1–4 are areas most likely to be forested, and one HCV criterion is that forest areas should be identified and protected if they are important for forest connectivity and/or the preservation of forest corridors.

Previous studies have examined the potential for HCV forest patches to support biodiversity (Lucey et al., 2017), but the contribution of current HCV forest patches to landscape connectivity has not been examined. Here, we meet this research need by evaluating the potential of forests in High Conservation Value Areas (HCVAs) to provide forest connectivity benefits. Our main aims are to: (a) determine the area and distribution of HCVAs in RSPO member‐held plantations in Borneo; (b) quantify the amount of 2015 forest cover within these HCVAs; and (c) examine the connectivity benefits of HCVAs for populations with different dispersal abilities. We assess landscape connectivity by using the Incidence Function Model (IFM; Hanski, 1994; Hodgson et al., 2011; Scriven, Hodgson, McClean, & Hill, 2015) to model range expansion of forest‐dependent populations across oil palm plantation landscapes. Hence, we define connectivity in our study as landscape colonization (i.e. the ecological process of range expansion), and so landscapes that are successfully colonized are deemed connected (e.g. see Scriven et al., 2015). We then quantify the connectivity benefits of HCVAs by comparing range expansion rates when HCVAs are simulated to be either present or absent. We test two hypotheses: (a) HCVAs containing more forest that are located in landscapes where HCVAs provide stepping‐stone patches generate greater connectivity benefits; and (b) connectivity benefits of HCVAs depend on population dispersal ability and forest cover within the wider landscape.

## 2 MATERIALS AND METHODS

### 2.1 HCVA and forest land‐cover data

**Figure 1:**
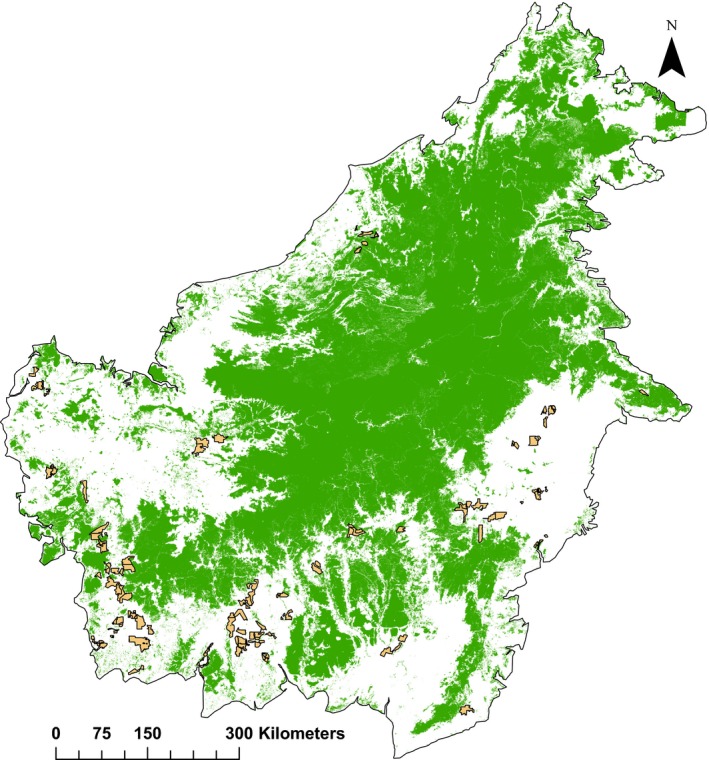
Map of Borneo showing location of 70 New Planting Procedure (NPP) assessment plantations (light orange shading) belonging to 28 Roundtable on Sustainable Palm Oil (RSPO) members. Distribution of forest cover (green shading; 30 m grid‐cell resolution) represents 2015 intact, logged and regrowth forest according to Gaveau et al. (2016)

Starting on 1 January 2010, the RSPO required that all members undertake the New Planting Procedure (NPP; RSPO, 2015), comprising assessments to be conducted prior to new oil palm developments, to prevent new plantings from negatively impacting areas of primary forest, HCV and fragile/marginal soils. Following the NPP assessment, auditors submit a report detailing where new plantings may take place to the RSPO for approval. We obtained the location of HCVAs by digitizing HCVA and plantation boundary maps from such NPP audit reports for 70 RSPO member‐held plantations in Borneo, including one in Sarawak, Malaysia and 69 across Kalimantan, Indonesia (Figure 1; also see Appendix S1 for digitization details). Around 50% of all 200 NPP assessments published by August 2018 occurred in Borneo (K.M. Carlson, unpubl. data, August 2018). Land‐cover data (30 m resolution) for 2015 were downloaded from the Atlas of Deforestation and Industrial Plantations in Borneo (https://www.cifor.org/map/atlas/; see Gaveau et al., 2016 for details). We combined intact, logged and regrowth forest land‐cover classes into a single class that we termed ‘forest’, and considered all other land‐cover categories as ‘non‐forest’. We aggregated these data to 90 m resolution by assigning each larger grid‐cell a value representing the number of the nine aggregated 30 m grid‐cells that contained forest, so that cell values ranged from zero (0% forest) to nine (100% forest). We chose 90 m resolution to ensure computationally feasible simulations while ensuring model sensitivity to the small area of HCVAs.

**Figure 2:**
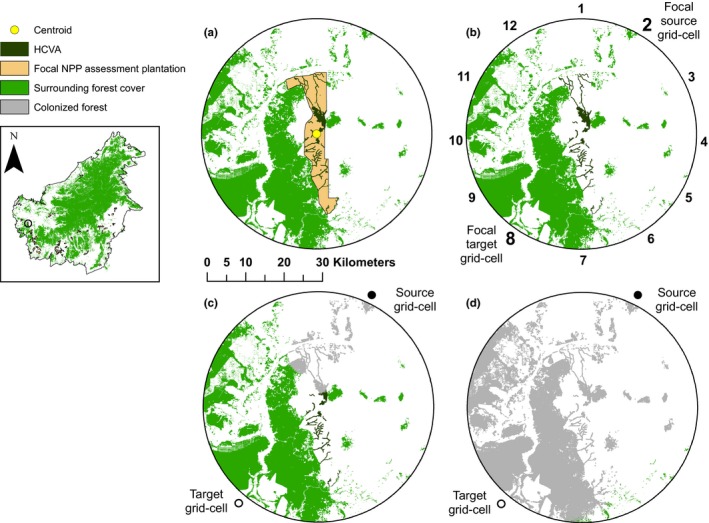
(a) Surrounding forest cover, High Conservation Value Areas (HCVAs) and estate area within a 30 km radius of an exemplar New Planting Procedure (NPP) assessment plantation in Kalimantan (Indonesian Borneo). The centre point (centroid) of the plantation is represented by a yellow circle. (b) An example plantation ‘landscape’ used to examine the connectivity benefits of HCVAs; numbers represent 12 different starting locations from which ‘source’ populations were seeded (i.e. forested 90 m grid‐cells that were occupied at the start of each simulation). Each source population needed to colonize a forested ‘target’ grid‐cell on the opposite side of the landscape. Hence, source population ‘2’ needed to colonize its target at location ‘8’ in less than 100 generations for the model simulation to be deemed successful. Thus, each number represents a single Incidence Function Model (IFM) simulation, and separate model run for each plantation. (c) Example simulation output whereby populations with 0.5 km dispersal did not colonize the target location within 100 generations (i.e. an ‘unsuccessful’ colonization). Colonized grid‐cells after 100 generations are shown in grey. (d) Example simulation output whereby populations with 3 km dispersal per generation successfully colonized the target grid‐cell within 100 generations (i.e. a ‘successful’ colonization). Inset map shows location of property in Kalimantan, Borneo. In this example, the plantation comprised only one spatially discrete estate and no other plantations included in this study fell within 30 km of the focal plantation centroid

Oil palm plantations often comprise several estates. In our dataset, individual estates within a single NPP assessment (subsequently termed a ‘plantation’) spanned distances of up to ~27 km (Figure S2 in Appendix S1). We assessed the area, core area, forest cover in 2015 and placement of HCVAs within these 70 plantations using ArcGIS version 10.4.1. Core area of HCVA patches (spatially discrete areas designated as HCV) was calculated by removing a buffer of 100 m from the edge of each patch (Lucey et al., 2017; also see Appendix S1 for additional details of geospatial statistics). In addition to HCVAs, many estates contained non‐HCVA forest cover within the plantation boundary. This forest could represent areas planned for development, given that oil palm producers undergoing the NPP have lands planned for oil palm plantings but have not yet commenced clearing. Moreover, in Indonesia, national law requires that plantation companies convert all arable concession lands, including currently forested areas, to agriculture (Republic of Indonesia, 2014). Hence, we removed all non‐HCVA forest found within the plantation boundaries for our connectivity analyses (823 km^2^ across all plantations). This equated to ~8% (823/9884 km^2^) of the total plantation area across the 70 plantations. To delimit plantation landscapes for our connectivity analyses and include all separate estates for any given NPP assessment plantation, we considered land‐cover within a 30 km radius (the plantation ‘landscape’) around the centre point (centroid) of each of the 70 plantations (Figure 2a; Figure S2 in Appendix S1). With this size of study landscape, we were able to assess the importance of HCVAs for connectivity in the context of the wider landscape, including habitat beyond the plantation boundary, over distances relevant to the types of species we were modelling.

### 2.2 Modelling the contribution of HCVAs to forest connectivity using the IFM

We examined the potential connectivity benefits of HCVAs using a patch‐based metapopulation model (IFM; Hanski, 1994). Our measure of connectivity was based on successful range expansion of populations across our 70 plantation landscapes, and we ran separate connectivity models for each plantation. We examined whether forest‐dependent populations with a range of dispersal abilities could successfully colonize forest networks within these plantation landscapes over multiple generations (see Hodgson et al., 2011; Scriven et al., 2015). The IFM examines habitat connectivity based on colonization and extinction dynamics, which are calculated by considering the size of forest patches, the distance to all surrounding forest patches, and species‐specific parameters such as dispersal and fecundity (Hanski, 1994; see Appendix S1 for IFM details).

For each of the 70 plantation landscapes, we simulated range expansion from ‘source’ to ‘target’ grid‐cells located on opposite sides of the landscape (Figure 2b; 12 replicates per landscape). All source grid‐cells were seeded with full forest cover, regardless of the forest fraction derived from the land‐cover data, to prevent source populations from going immediately extinct. Each simulation was terminated once an individual colonized a target grid‐cell (a ‘successful’ colonization; see Figure 2d), or after 100 generations if no individuals reached the target grid‐cell (an ‘unsuccessful’ colonization; Figure 2c). Individuals could move across the plantation landscape in any direction but were constrained to reproduce only within forest. We excluded source and target grid‐cells over water for six plantations near the coast.

#### 2.2.1 Testing connectivity benefits of HCVAs according to the amount of forest they contain

To examine the benefits of HCVAs for forest connectivity, we ran IFMs under three different scenarios, assuming HCVAs were (a) absent and contained no forest cover (‘no forest’); (b) present with current (2015) forest cover (‘current forest’); or (c) present with full (100%) forest cover (‘full forest’). The no forest scenario provides a counterfactual that assumes that without RSPO membership, companies would not conserve HCVAs, but plant these areas with oil palm. The current forest cover scenario represents our best estimate of the current contribution of HCVAs to connectivity. The full forest scenario assumes that all HCVAs are reforested and represents the greatest potential contribution of HCVA designation to connectivity. Since not all HCVAs contain forest or protect biodiversity (e.g. graveyards may be designated because of their cultural value), the full forest cover scenario is likely an overestimate of the benefits of the RSPO for connectivity (see Appendix S1 for further details).

#### 2.2.2 Modelling impacts of dispersal ability on HCVA connectivity

We examined how different assumptions of population dispersal ability affected our measures of forest connectivity, by varying *α* (alpha), the slope of a negative exponential dispersal kernel within the IFM. This alpha value was inferred by assuming that 5% of individuals within the population could go further than the stated maximum (see Hodgson et al., 2011). We examined five dispersal values corresponding to maximum dispersal distances of 0.5, 1, 3, 5 and 10 km per generation (see Appendix S1). Thus, our model examined different types of populations, ranging from relatively sedentary species (e.g. flightless insects), to relatively mobile vertebrates (e.g. birds or bats). We present results only for population densities of 20 individuals per forested ha (representing winged insects; e.g. see Benedick et al., 2006) because IFM outputs were generally similar when we ran models with alternate population density values (Appendix S1; also see Scriven et al., 2015).

### 2.3 Analyses of model outputs

We ran connectivity models simulating range expansion across 70 plantations, from 12 different starting locations per planation (Figure 2b) for three HCVA scenarios and five dispersal abilities (i.e. 15 treatment combinations in a fully‐factorial design). We used a Generalized Additive Model (GAM: binomial logistic regression; R package *mgcv*: see Wood, 2011; Appendix S1 for more details) to examine forest connectivity according to the probability of successful colonizations across 70 plantation landscapes. In this model, the dependent variable was a two‐column matrix that represented the number of successful and unsuccessful colonizations across each plantation landscape, from the 12 replicates (Figure 2b). To prevent each replicate from being treated as independent, we weighted each row of data by the reciprocal of the total number of replicate IFM runs for each plantation (e.g. 1/12). We included dispersal ability and HCVA forest cover scenario as categorical predictor variables. To examine the importance of forest (defined in Section 2.1) within the wider landscape on plantation connectivity, our model also included the area of forest cover within each landscape (i.e. outside the focal plantation, but within a 30 km radius of each plantation centre; see Figure 2a). Finally, we included an interaction between the latitude and longitude of each plantation centre (Wood, 2006). The interaction was fitted as a nonlinear (smooth) term selected at an optimal level of complexity by the fitted algorithm. By modelling spatial dependence in the systematic part of the model, we were able to account for spatial autocorrelation in the model residuals, determined by inspecting correlograms (see Dormann et al., 2007). We kept all variables in the GAM to examine their relative importance on forest connectivity, and we ran the model using a logit link and binomial errors. To examine the importance of HCVA forest cover scenario, irrespective of dispersal ability, we ran a second GAM without dispersal ability included as a predictor variable, but kept all other model parameters the same. Finally, to examine the robustness of our model outputs, we reran the full analysis using a Generalized Linear Mixed Model (GLMM; Table S1 in Appendix S1 and Figure S3 in Appendix S1), but our main conclusions were similar across these two models, and so we only present findings from the GAM analysis in the main text. All statistical analyses were carried out in R version 3.4.0.

## 3 RESULTS

### 3.1 Size and amount of forest in HCVAs

**Figure 3:**
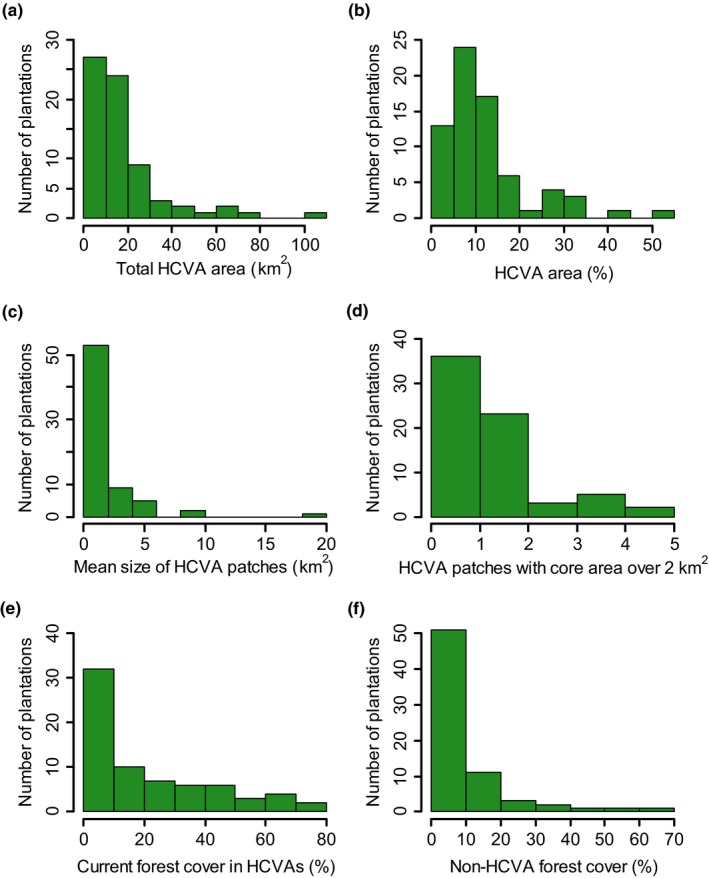
Histograms showing (a) total High Conservation Value Area (HCVA) area (km^2^) per plantation, (b) percentage of each plantation deemed HCVA, (c) mean size (km^2^) of HCVA patches per plantation, (d) number of HCVA patches with a core area greater than 2 km^2^ per plantation, (e) percentage of 2015 forest cover within HCVAs per plantation, and (f) percentage of each plantation covered by non‐HCVA forest

The 70 NPP plantations ranged in size from 10 to 547 km^2^ (mean = 141, *SD* ± 81 km^2^). In these plantations, on average HCVAs comprised ~12% of the total plantation area (*SD* ± 10%; ranging from 0.6% to 53%; Figure 3b). The mean area of individual HCVA patches (*N* = 1,040), was 1.2 km^2^ (*SD* ± 4.4) (Figure 3c) and on average HCVAs were only about one‐fifth forested (mean forest cover in HCVAs across the 70 plantations = 21%, *SD* ± 22%, Figure 3e). Across all HCVAs, HCV types important for biological diversity and ecosystem services were the most extensive in terms of both area and forest cover, and were present in all plantations (Table S2 in Appendix S2).

### 3.2 Connectivity benefits of HCVAs

**Figure 4:**
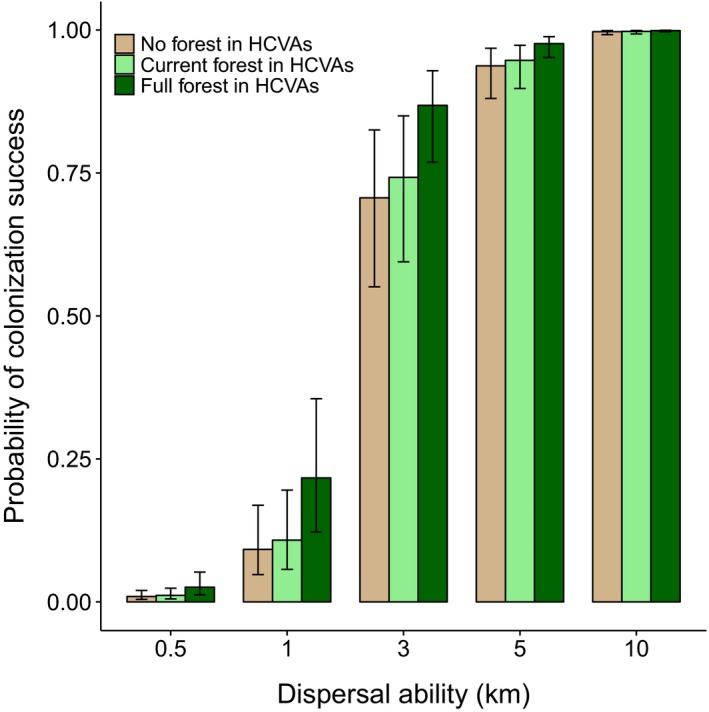
Probabilities of successful colonization of oil palm landscapes across High Conservation Value Area (HCVA) scenarios for populations with different dispersal abilities: brown shading = no forest cover scenario, light green shading = current (2015) forest cover scenario, and dark green shading = full forest cover scenario. Probabilities are predicted values from the Generalized Additive Model (binomial logistic regression) where all covariates are held constant (i.e. at their mean values). Bars represent standard errors

There were few connectivity benefits provided by HCVAs under 2015 forest cover (i.e. ‘current forest’ scenario). Compared to landscapes with no HCVAs (i.e. ‘no forest’ scenario) current HCVAs improved connectivity by only ~3% for all populations (i.e. across all dispersal distances; Figure S4 in Appendix S2; Table S3 in Appendix S2). When dispersal ability was considered, HCVAs with current forest cover had the greatest relative connectivity benefits for populations with poor dispersal abilities (0.5 km). For these types of species, landscapes with current forest cover in HCVAs were on average 1.2 times better connected than landscapes with no HCVAs, hence a ~20% improvement to connectivity (Figure 4; Table S4 in Appendix S2). Nevertheless, since poor dispersers rarely colonized plantation landscapes successfully regardless of HCVA forest cover, the absolute improvement to connectivity was small, increasing from a probability of colonization success of .0095 with no HCVA forest cover to .0114 with current forest cover, an overall improvement of just .0019 (Figure 4).

Fully reforested HCVAs (i.e. ‘full forest’ scenario) provided greater connectivity benefits than did HCVAs with current forest cover. Overall, irrespective of dispersal ability, the relative improvement to connectivity provided by reforested HCVAs compared to HCVAs with no forest cover was ~16% (Figure S4 in Appendix S2; Table S3 in Appendix S2). When dispersal ability was considered, the greatest percentage improvement to connectivity with HCVA reforestation occurred for populations with poor to intermediate dispersal abilities (Figure 4; Table S4 in Appendix S2). Specifically, populations with 0.5, 1 and 3 km dispersal abilities were on average 2.7, 2.4 and 1.2 times more likely to successfully colonize plantation landscapes with full forest cover in HCVAs, compared to landscapes with no HCVAs, respectively (Figure 4). Despite HCVA reforestation, absolute connectivity benefits were small for the poorest dispersers, as most populations were still unable to successfully colonize plantation landscapes (Figure 4). These findings were relatively insensitive to variation in population density, although reforested HCVAs may have greater absolute connectivity benefits for the very poorest dispersers if their population densities are high (Figure S1 in Appendix S1). Absolute connectivity benefits following HCVA reforestation were therefore greatest for populations with 1 and 3 km dispersal abilities, for which the probability of successful colonization increased by 0.13 and 0.16, respectively (Figure 4). For populations with 5 and 10 km dispersal abilities, both relative and absolute improvements to connectivity were low because the number of successful colonizations was already high (Figure 4).

### 3.3 Surrounding forest cover and landscape connectivity

**Figure 5:**
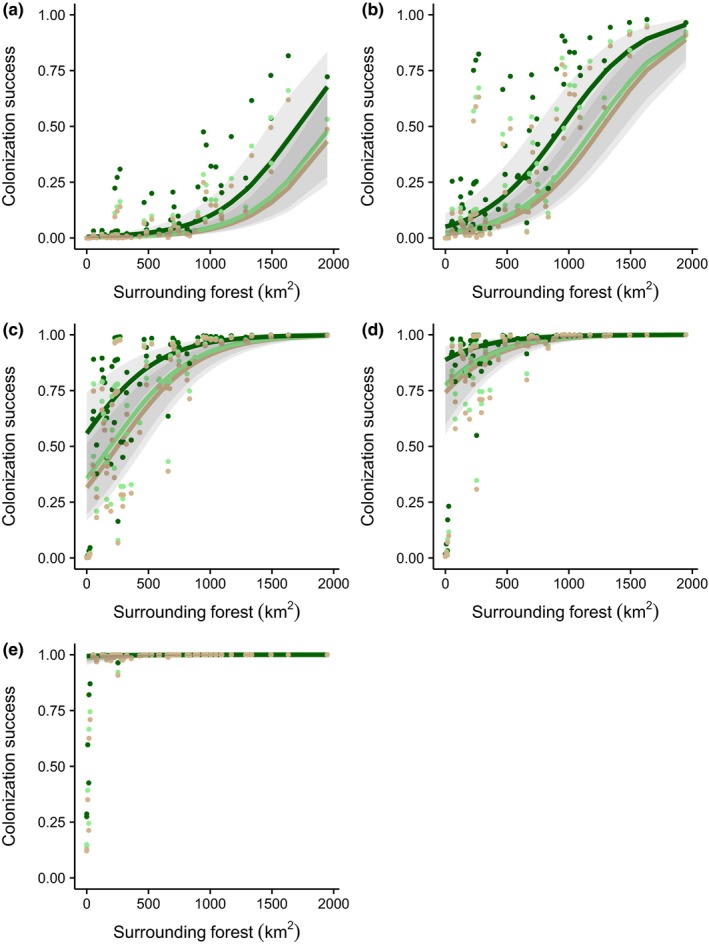
Relationship between the probability of successful colonization of oil palm landscapes and the area of forest cover surrounding each plantation for populations with (a) 0.5 km, (b) 1 km, (c) 3 km, (d) 5 km and (e) 10 km dispersal abilities. Points and lines are colour coded to represent landscapes with different amounts of forest cover in High Conservation Value Areas (HCVAs; i.e. HCVA scenarios): brown shading = no forest cover, light green shading = current (2015) forest cover, and dark green shading = full forest cover. Points represent predicted values from the Generalized Additive Model (binomial logistic regression) and lines represent model fit (i.e. when all other predictor variables are at their mean values) for each HCVA scenario. Grey shading represents standard errors around model fit lines

Across all HCVA scenarios, the probability of successfully colonizing plantation landscapes increased with dispersal ability and was highest in landscapes with more surrounding forest cover (Figures 4 and 5; Table S4 in Appendix S2). For populations with 0.5 km dispersal ability (i.e. representative of very sedentary species) the probability of successful colonization was relatively low regardless of HCVA scenario, but increased with higher levels of surrounding forest cover (Figure 5a). Conversely, for populations with 5 to 10 km dispersal abilities (i.e. representative of very mobile species), the probability of successfully colonizing plantation landscapes was always high, except for extremely isolated plantations with very low levels (i.e. <100 km^2^) of surrounding forest cover (Figure 5d,e).

## 4 DISCUSSION

### 4.1 Characteristics of HCVAs

High Conservation Value Areas in oil palm plantations comprised around 12% of the total plantation area, and so have the potential to make an important contribution to remaining forest cover in oil palm landscapes. Furthermore, almost half of all plantations contained at least one HCVA patch that had a core area larger than 2 km^2^ (200 ha), which may provide substantial biodiversity benefits compared to oil palm (Lucey et al., 2017), and have the potential to maintain populations of forest species. Conservation of large tracts of high‐quality forest habitat is important for population persistence in human‐modified landscapes (e.g. see Edwards, Fisher, & Wilcove, 2012; Lucey et al., 2017), and so small HCVAs may be unable to support viable populations of forest‐dependent species unless they are well‐connected to other forested areas. However, our results suggest that if well positioned between large tracts of forest, smaller HCVAs may act as ‘stepping stones’ to facilitate movement across fragmented landscapes (Hodgson, Wallis, Krishna, & Cornell, 2016).

High Conservation Value Areas will provide the largest benefits for both biodiversity and connectivity if they contain high‐quality forest (Scriven et al., 2015; Tawatao et al., 2014), but HCVAs in our study were only 21% forested, including intact, logged and regrowth forest. Our estimates of forest cover are likely to be conservative, as they may not include all disturbed and severely burned forest areas (Gaveau et al., 2016), but provide an indication of how much high‐quality forest is conserved within HCVAs as of 2015. HCVAs identified in plantations before any plantation development activities had commenced (i.e. completely new developments after 2010) contained a higher percentage forest cover than HCVAs in ongoing plantings (Appendix S1 and S3). Nevertheless, across all plantations, forest cover in HCVAs was low, and so there is a pressing need to restore forest habitats within existing HCVAs.

### 4.2 Benefits of HCVAs for connectivity

Our results suggest that HCVAs currently provide little benefit for connectivity, although landscapes with HCVAs were still up to 1.2 times better connected than landscapes without HCVAs for some populations. Connectivity improved (up to 2.7 times better) for all populations when HCVAs were reforested compared to landscapes with no HCVAs. However, for poor dispersers with very high population densities, connectivity benefits of reforested HCVAs may be even higher (Figure S1 in Appendix S1). As HCV types 5 and 6 are put in place to protect community needs and cultural values rather than biodiversity (see https://www.hcvnetwork.org/), it is likely that these results are somewhat optimistic, as reforestation may not be feasible or support the values that led to HCVA designation. Also, our ‘no forest’ scenario is not a perfect counterfactual of the benefits of certification, as we do not know how much forest remains in non‐RSPO plantations.

We used the IFM (Hanski, 1994) to quantify connectivity because this measure represents a key ecological process (range expansion), which incorporates ecological realism (e.g. metapopulation dynamics) and so produces more ecologically‐relevant outcomes compared to simpler approaches. Our results are comparable to those of more standard connectivity metrics (e.g. least‐cost models; see Appendix S4), but our IFM approach enables us to examine whether habitat networks of conservation set‐asides will allow species to colonize and persist over multiple generations (Hodgson et al., 2011). There is a need to develop modelling approaches that assess the resilience of ecological networks and that go beyond classic landscape connectivity estimates and incorporate ecological outcomes (Isaac et al., 2018). Our approach is therefore an improvement on standard connectivity metrics, but does not include parameters such as reproductive strategy or dispersal phase that are often included in more complex Individual Based Models (IBMs; e.g. see Synes et al., 2015), which are more flexible and predictive than IFMs, but also more computationally intensive. More research is needed to better understand the resilience of habitat networks and identify where connectivity losses are most critical.

### 4.3 Role of dispersal on connectivity benefits

In landscapes with both current and full forest cover in HCVAs, absolute connectivity benefits were greatest for populations with intermediate dispersal abilities (1–3 km dispersal; representative of fairly mobile species such as forest‐dependent butterflies or small sub‐canopy birds). Despite high relative connectivity benefits (i.e. percentage improvement), HCVAs provided few absolute connectivity benefits (i.e. change in probability) for extremely sedentary populations, such as weak‐flying, insects (e.g. see Malohlava & Bocak, 2010) that disperse less than 0.5 km per generation. These types of species are likely unable to cross non‐forest areas, and so may require continuous tracts of forest to move across plantation landscapes. HCVAs also provided little connectivity benefit for extremely mobile species dispersing more than 5 km per generation because landscapes are nearly always connected for these species (e.g. large birds or bats; see Corlett, 2009; Figure 4). In our connectivity models, we assumed that populations of forest species could leave forested areas and disperse across plantation matrices. In reality, little research has examined the permeability of oil palm plantations for forest‐dependent species, which may be confined to forest habitats if they are unable to cross forest‐plantation edges (Scriven et al., 2017).

### 4.4 Influence of the wider landscape on connectivity benefits of HVCAs

The availability of forest in the surrounding landscape varied considerably, and plantations with more surrounding forest were better connected for all types of forest populations. Whilst we did not explicitly explore the relationship between HCVA size and the connectivity benefits of HCVAs, it is likely that even large HCVAs provide little connectivity benefit if they are too isolated from other forested areas in the wider landscape (Figure S5 in Appendix S2). Similarly, HCVAs may also provide few additional connectivity benefits if located within reasonably intact landscapes that are already well‐connected. HCVAs are therefore likely to provide the most connectivity benefits in landscapes with a patchy mix of forest and non‐forest areas, dependent on the specific location of HCVAs in relation to surrounding forest (i.e. the intermediate landscape‐complexity hypothesis; see Tscharntke et al., 2012; Figure S5 in Appendix S2).

### 4.5 Conservation implications and recommendations

Almost half of all plantations we studied contained at least one HCVA patch large enough to support forest‐dependent species (i.e. with a core area >2 km^2^) (Lucey et al., 2017), but these HCVAs may not contain good quality forest, which is needed for maintaining tropical biodiversity (Tawatao et al., 2014). Many of the HCVAs we studied had low forest cover, and we strongly recommend active management by plantation companies to improve forest extent and quality, such as enrichment planting (Yeong, Reynolds, & Hill, 2016). Improving the quality of HCVAs may not only benefit landscape connectivity but also provide important ecosystem services such as pollination (Kormann et al., 2016) and prevention of soil erosion (Dislich et al., 2017). To incentivize oil palm growers to enhance forest quality, we recommend modification of HCV guidance documents and the RSPO's Principles and Criteria (P&C; see RSPO, 2018) to require restoration of degraded HCVAs. Current RSPO guidelines are not prescriptive about strategies for maximizing HCVA connectivity in relation to the wider landscape (e.g. for P&C 7.12; RSPO, 2018). We therefore recommend that if large (i.e. with a core area >2 km^2^), isolated HCVAs are identified during HCV assessments, then provision should be made to reconnect these areas via restoration of the intervening plantation matrix. Hence, future revisions to the standard should explicitly ensure that large, isolated HCVAs are reconnected to other tracts of forest such as public protected areas, community‐managed forests (Santika et al., 2017), and/or production forests, which can maintain high levels of biodiversity (Edwards et al., 2011).

By May 2019, following 3–4 years of further NPP assessments since our cut‐off in 2015, an additional 40 NPP plantations had been assessed in Borneo (https://www.rspo.org/certification/new-planting-procedure/public-consultations). As NPP regulations have remained the same since 2010 (RSPO, 2015) we would not expect any HCVAs within these additional NPP plantations to be different from those in our analyses. Nevertheless, the incorporation of the Assessor Licencing Scheme (ALS) into the NPP in 2015 (see https://hcvnetwork.org/als/) may have had positive impacts on forest connectivity if more forest was designated as HCVA. Additionally, in November 2018, the RSPO revised its P&C and incorporated a zero‐deforestation policy (P&C 7.12; RSPO, 2018) via the inclusion of the High Carbon Stock (HCS) approach. The requirement for connectivity is now more implicit in the HCS Approach Toolkit (i.e. via the HCS Forest Patch Analysis Decision Tree; Rosoman, Sheun, Opal, Anderson, & Trapshah, 2017) and the HCV Common Guidance document (e.g. in relation to HCV 2 for ensuring intact forest landscapes; Brown, Dudley, Lindhe, Muhtamen, & Stewart, 2013). These changes are expected to increase the amount of forest set‐aside in new plantings (RSPO, 2018), improving biodiversity (Deere et al., 2018) and connectivity in RSPO‐dominated landscapes. We recommend that the RSPO publish digitized maps of HCV/HCS areas, to provide opportunities for maintaining connectivity of HCVAs at landscape scales and facilitate cooperation between neighbouring RSPO member plantations. However, jurisdictional approaches including designation of HCVAs across districts or states (Pacheco, Hospes, & Dermawan, 2017) may be needed to fully realize the potential for linking HCVAs with forest outside the focal plantation. We conclude that improvements to the RSPO standard will likely improve the connectivity benefits of HCVAs, but more research is needed at landscape scales to test these benefits in the long term.

## AUTHOR'S CONTRIBUTIONS

The specific contributions are as follows: S.A.S., J.K.H., K.M.C. and J.M.L. conceived and designed the research; J.A.H., S.A.S., C.J.M. and J.K.H. conceived and developed the connectivity simulations, which were run by S.A.S. and C.J.M.; K.M.C., S.A.S. and R.H. conceived and oversaw HCVA digitization; S.A.S. analysed the data, with input from J.A.H., R.H. and C.J.M.; and S.A.S. drafted the manuscript. All authors provided manuscript modifications and gave approval for publication.

## Data Availability

Data available via the Dryad Digital Repository: https://doi.org/10.5061/dryad.600vs50 (Scriven et al., 2019).
