# Supplementary material for: Testing the benefits of conservation set‐asides for improved habitat connectivity in tropical agricultural landscapes
Source: J Appl Ecol. 2019 Aug 19;56(10):2274–85. doi: 10.1111/1365-2664.13472 (PMC6853203; doi:10.1111/1365-2664.13472)
Supplement: Supplementary file 1 [file JPE-56-2274-s001.docx]

**­Testing the benefits of conservation set-asides for improved habitat connectivity in tropical agricultural landscapes**

**Supporting information**

**Appendix S1. Additional methodological details**

**Digitization of High Conservation Value Areas (HCVAs) in New Planting Procedure (NPP) assessment plantations**

New Planting Procedure assessments were obtained online from the Roundtable on Sustainable Palm Oil (RSPO) (https://www.rspo.org/) for oil palm growers in Borneo. Maps depicting oil palm plantation boundaries and HCVAs were extracted from these NPP assessments and were georeferenced and digitized in ArcMap version 10.4.1. While RSPO assessment reports for certification may include maps of HCVAs, the quality of these maps is generally insufficient for accurate digitization. Hence, we focused our connectivity analyses solely on NPP assessments. New Planting Procedure assessment plantations (termed ‘plantations’) included both completely new developments and ongoing plantings. As the RSPO’s NPP was initiated in 2010, completely new developments represent lands slated for land preparation and planting in 2010 or later (i.e. they had not yet been cleared and planted when the assessment was conducted), whilst ongoing plantings represent lands where planting was initiated before 2010. Thus, completely new developments were plantations that contained HCVAs and were identified as part of the NPP process (i.e. that underwent a HCV assessment before development commenced).

We digitized boundaries and HCVAs from 70 randomly-selected NPP assessment reports from 28 RSPO member companies (see Figure 1 in main text). Where audit report quality was sufficient, we excluded water bodies (i.e. rivers that were classified as HCVAs) from our connectivity analyses. We did this because we were primarily interested in examining the connectivity of terrestrial HCVAs and inclusion of water bodies would overestimate the connectivity benefits of HCVAs in the ‘full forest’ scenario. These separate features could only be identified when water bodies were large and image quality was high, which meant that the size of the water bodies excised from the HCVA dataset varied. Small streams could often not be distinguished from surrounding riparian HCVAs, and so were included as HCVAs in our analyses. Therefore, in the reforested scenario, such water bodies were converted to forest grid-cells at a 30 m resolution.

Where possible, HCVA polygons were classified by HCV type (e.g. 1 to 6; see https://www.hcvnetwork.org/). We included all HCVA types and all HCV management areas in our analyses because HCVA classifications were not available for all plantations. Importantly, HCVAs – including types 5 and 6 – are not always designated for their forest cover. However, inclusion of these HCVA types is unlikely to severely influence our results. First, types 5 and 6 could often not be digitized because of their very small size, so they may be under-represented in our dataset. Second, there are also some instances where these HCVA types may be forested (i.e. for timber extraction, fuel resources, clean water protection and sacred forest sites).

**Geospatial statistics:**

For each NPP assessment plantation digitized (*N* = 70), we calculated the total area of all HCVAs, the percentage of the total plantation area designated as HCVA, and the percentage forest cover within HCVAs. We also examined the average size of all spatially discrete HCVA patches across plantations and calculated the ‘core area’ of each HCVA patch. Core area of HCVA patches was calculated by removing a buffer of 100 m, which we assume is the distance over which most edge effects cause detrimental impacts (Laurance et al., 2002), from the edge of each patch (see Lucey et al., 2017). In 44 plantations, information on HCVA type was available, and so we calculated the area and percentage forest cover across different HCVA types for those plantations (Table S2).

**The Incidence Function Model (IFM):**

The IFM (see Hanski, 1994) is based on the assumptions that (a) extinction probability (i.e. the likelihood that a population goes extinct from any given habitat patch) is inversely related to population size and habitat patch area (i.e. the number of 30 m forest grid-cells within an aggregated 90 m grid-cell), and (b) the probability of patch colonization is positively related to patch connectivity, whereby the connectivity is a function of the distance to other occupied forest cells and the amount of forest they contain (Hanski, 1994). The connectivity (S_i_) of each patch (a spatially discrete forested grid-cell(s)), (*i*), is defined as:

$$A_{i}\frac{R\alpha^{2}}{2\pi}\sum_{j\neq i} {p_{j}A}_{j}e^{{-\alpha d}_{ij}}$$

where *A* = area of habitat (km^2^) in forested grid-cell *i* or *j*, *R* = population density (number of emigrants (individuals) produced per generation per occupied 90 m grid-cell), *α* = slope of a negative exponential dispersal kernel, *p_j_* = occupancy of *j* (1 if grid-cell *j* is occupied, 0 if not) and *d_ij_* is the Euclidean distance between the centre of grid-cells *i* and *j*. To estimate the carrying capacity of each grid-cell, the amount of forest (i.e. the number of 30 m grid-cells) within each 90 m grid-cell is multiplied by the population density. The extinction probability is subsequently 1/carrying capacity of each 90 m grid-cell at each generation, except within source cells where it was set to zero (see Hodgson et al., 2011, Scriven, Hodgson, McClean, & Hill, 2015, for further details).

**Dispersal parameters:**

We examined five dispersal values corresponding to maximum dispersal distances of 0.5, 1, 3, 5 and 10 km per generation. The lowest dispersal ability (0.5 km) was chosen to represent an extremely sedentary species such as a flightless, or weak-flying, insect that may require intact forest (e.g. see Malohlava & Bocak, 2010), whilst the intermediate dispersal abilities (1 and 3 km) were chosen to represent fairly mobile species, such as large rainforest butterflies (e.g. see Marchant et al., 2015) or small sub-canopy birds. We also included dispersal abilities that most represent highly mobile populations that can disperse more than 5 km per generation. These are most representative of species such as large birds or bats, which may be able to disperse across large gaps that separate remaining forest habitats (e.g. see Corlett, 2009). We ran our models for 100 generations, a value chosen to allow most populations with poor dispersal ability sufficient time to colonize each landscape assuming it was entirely forested. Hence, colonization time is a function of the size of the landscape (e.g. 60 km diameter) and the population’s dispersal ability (e.g. 0.5 km). Setting a fixed number of generations (rather than years) allowed us to infer the time it would take for different types of ‘species’ to colonize plantation landscapes. For example, a species with one generation per year would have up to 100 years to colonize the landscape.

**Population density parameters:**

Our IFM outputs presented in the main text are for populations with 20 individuals per forested ha. However, we ran additional IFMs with population density set much lower and higher than the value in the main text, to 2 and 200 individuals per forested ha. These represent plausible values for different types of species (i.e. mammals with very low population densities and invertebrates with much higher density values) and allowed us to examine the effect of changing this parameter on our findings. We reran the IFMs for populations with 0.5 and 3 km dispersal abilities for all our HCVA scenarios. These dispersal distances were chosen specifically because reforestation of HCVAs had the greatest benefit for poor to intermediate dispersers (0.5-3 km per generation), whilst range expansion of highly mobile species was relatively unaffected by habitat loss (i.e. nearly all model simulations were successful for populations with >5 km dispersal abilities; see main text and Figure 4). Hence, we ran additional models for three HCVA scenarios, two population densities and two dispersal treatments (i.e. 12 treatment combinations), for all 70 plantation landscapes (Figure S1).

For populations with 0.5 km dispersal ability and 2, 20 and 200 individuals per forested ha, landscapes with reforested HCVAs were 1.09, 1.19 and 1.63 times better connected than in landscapes with no HCVAs, respectively, and so reforested HCVAs may have greater connectivity benefits for very poor dispersers with high population densities (Figure S1). For populations with 3 km dispersal ability and 2, 20 and 200 individuals per forested ha, landscapes with reforested HCVAs were 1.33, 1.25 and 1.19 times better connected than in landscapes with no HCVAs, respectively, and so HCVA benefits were similar across our wide range of population density estimates (Figure S1). Note that to enable comparison, improvement values presented here were calculated from raw IFM output probabilities and are not predicted probabilities from the GAM, where all covariates were held constant (as presented in the main text). These additional analyses for low and high population densities did not alter our main findings and conclusions, which are robust to different population density parameter values.

**Analyses of model outputs:**

The statistical relationship between the predictor variables and the probability of successful colonizations across plantation landscapes was modelled using Generalized Additive Models (GAMs). Generalized Additive Models are a class of statistical regression that allow for non-linear relationships by extending Generalized Linear Models (GLMs) and incorporating a semiparametric ‘smooth term’. The complexity of the non-linear relationship for each predictor variable is described by the effective degrees of freedom (edf) of the smooth term, and the form and complexity of the smooth term is a trade-off between the better fit of complex curves and the predictive ability of the model. Hence, the interaction between latitude and longitude (see main text) was selected at an optimal level of complexity, which is a trade-off between goodness of fit and the predictive accuracy of simpler functional relationships (see Scroggie & Clemann, 2009; Wood, 2006). To examine the robustness of our GAM outputs, we reran the same overall analysis using a Generalized Linear Mixed Model (GLMM; binomial logistic regression; R package *lme4*: see Bates, Mächler, Bolker, & Walker, 2015). In this model, the dependent variable was again a two-column matrix that represented the number of successful and unsuccessful colonizations across each plantation landscape, from the 12 replicates (see Figure 2b in main text), and we included HCVA scenario as a categorical predictor. To ensure that the model converged, we included dispersal ability as a continuous predictor with an orthogonal polynomial transformation. The area of surrounding forest cover within a 30 km radius of the plantation centre was also included as a continuous predictor, but the geographic coordinates (i.e. latitude and longitude) of each plantation centre were not included in the GLMM. Instead, in order to account for spatial autocorrelation in the model residuals, plantations were assigned into 10 clusters (or groups) depending on the specific spatial location of their plantation centroid, and plantation cluster was included as a random factor in the model. Plantation identity (i.e. a unique number between 1-70 assigned to each plantation) was subsequently nested within cluster. We kept all variables in the GLMM, to examine their relative importance on connectivity, and we ran the model using a logit link and binomial errors. Model outputs were comparable with the results of the GAM analysis presented in the main text (see Table S1 for model coefficients and Figure S3 for output probabilities of successful colonizations across HCVA scenarios and dispersal abilities).

**Table S1. Outputs from the Generalized Linear Mixed Model (GLMM; binomial logistic regression) determining the effects of dispersal ability (*N* = 5), High Conservation Value Area (HCVA) forest cover scenario (*N* = 3) and amount of surrounding forest cover (km^2^) on the probability of successful colonization for 70 plantation landscapes.**

| **Random effects** | **Variance** | **SE** |  |  |
| --- | --- | --- | --- | --- |
| Plantation ID: Plantation cluster | 0.4057 | 1.401 |  |  |
| Plantation cluster | 3.877 | 1.969 |  |  |
| **Fixed effects** | **Estimate** | **SE** | ***z* value** | ***P*** |
| Intercept | 0.4057 | 0.6674 | 0.61 | 0.543 |
| HCVA Scenario 2 (current forest) | 0.1992 | 0.08564 | 2.33 | 0.020 |
| HCVA Scenario 3 (full forest) | 1.112 | 0.08832 | 12.59 | <0.0001 |
| poly (Dispersal, 2) 1 | 121.2 | 3.321 | 36.48 | <0.0001 |
| poly (Dispersal, 2) 2 | -36.65 | 1.844 | -19.87 | <0.0001 |
| Surrounding forest (km^2^) | 2.420 | 0.2284 | 10.60 | <0.0001 |

**(a)**

**(b)**

**Figure S1. Probabilities of successful colonization of oil palm landscapes across High Conservation Value Area (HCVA) forest cover scenarios for populations with different population densities (representing the number of individuals per forested ha). Brown shading = no forest cover scenario, light green shading = current (2015) forest cover scenario, and dark green shading = full forest cover scenario, for (a) 0.5 km and (b) 3 km dispersal abilities. Probabilities are calculated from raw data and bars represent standard errors.**

**
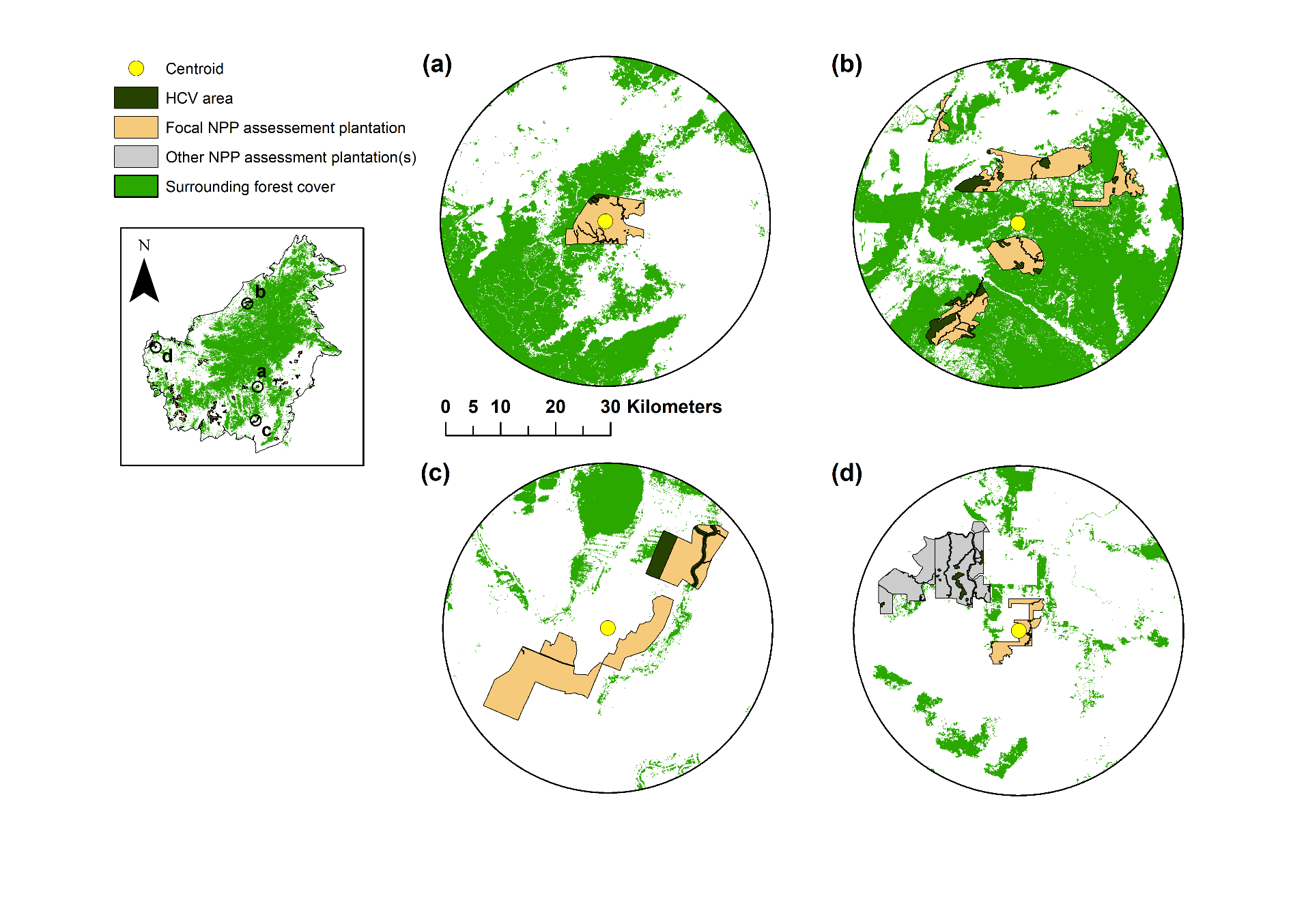
**

**Figure S2. Surrounding forest cover, High Conservation Value Areas (HCVAs) and estate area within 30 km of example New Planting Procedure (NPP) assessment plantations. The centre point (centroid) of the plantation is represented by a yellow circle. New Planting Procedure assessment plantation examples comprise: (a) one single estate, (b) and (c) multiple estates, and (d) one single estate that falls within 30 km of other NPP assessment plantation estates. These plantation ‘landscapes’ were used to examine the connectivity benefits of HCVAs (see Figure 2 in main text for further details).**

**Figure S3. Probabilities of successful colonization of oil palm landscapes across High Conservation Value Area (HCVA) forest cover scenarios for populations with different dispersal abilities: brown shading = no forest cover scenario, light green shading = current (2015) forest cover scenario, and dark green shading = full forest cover scenario. Probabilities are predicted values from the Generalized Linear Mixed Model (GLMM; binomial logistic regression) where all covariates are held constant (i.e. at their mean values). Bars represent standard errors for fixed effect uncertainty.**

**Appendix S2. Additional results**

**Table S2. Summary statistics and standard deviation (SD) calculated for different types of High Conservation Value Areas (HCVAs) across Roundtable on Sustainable Palm Oil (RSPO) New Planting Procedure (NPP) assessment plantations in Borneo.**

| **HCVA type** | **Number of plantations with HCVAs present (%) (*N* = 70)** | **Average total HCVA area (km^2^) across plantations (*N* = 44)^b^** | **Total HCVA area (km^2^) across plantations (*N* = 44)^b^** | **Average forest cover (%) across plantations (*N* = 44)^bc^** |
| --- | --- | --- | --- | --- |
| **1: Species diversity** | 100 | 12.6 (± 12.4) | 553 | 26 (± 28) |
| **2: Landscape-level ecosystems** | 49 | 3.6 (± 8.2) | 159 | 27 (± 30) |
| **3: Ecosystems and habitats** | 49 | 5.6 (± 15.5) | 248 | 31 (± 35) |
| **4: Critical ecosystem services** | 100 | 15.5 (± 19.3) | 682 | 21 (± 23) |
| **5: Community needs** | 47^a^ | 2.8 (± 6.3) | 122 | 19 (± 23) |
| **6: Cultural values** | 50^a^ | 1.5 (± 5.3) | 67 | 15 (± 22) |

^a^ The number of plantations containing HCVA types 5 and 6 may be underestimated, as these areas could often not be digitized because of their small size. These areas can represent sacred trees, graveyards, wells or other small features that are important for local communities.

^b^ These values have been calculated across the 44 plantations for which HCVAs could be classified by type. Note that many HCVAs are of more than one type and so occur across multiple categories; hence values are not additive.

^C^ Forest cover derived from Gaveau et al. (2016).

**Table S3. Outputs from the Generalized Additive Model (GAM; binomial logistic regression) determining the effects of High Conservation Value Area (HCVA) forest cover scenario (*N* = 3) and amount of surrounding forest cover (km^2^) on the probability of successful colonization for 70 plantation landscapes.**

| **Parametric (linear) terms** | **Estimate** | **SE** | ***z* value** | ***P*** |
| --- | --- | --- | --- | --- |
| Intercept | -0.5230 | 0.1876 | -2.787 | 0.0053 |
| HCVA Scenario 2 (current forest) | 0.0631 | 0.1659 | 0.381 | 0.7034 |
| HCVA Scenario 3 (full forest) | 0.357 | 0.1683 | 2.122 | 0.0339 |
| Surrounding forest (km^2^) | 0.0013 | 0.0003 | 4.822 | <0.0001 |
| **Smoothed (non-linear) terms** | **edf** | **Ref.df** | **Chi.sq** | ***P*** |
| Latitude, Longitude (interaction) | 15.69 | 19.98 | 61.72 | <0.0001 |

**Table S4. Outputs from the Generalized Additive Model (GAM; binomial logistic regression) determining the effects of dispersal ability (*N* = 5), High Conservation Value Areas (HCVA) forest cover scenario (*N* = 3) and amount of surrounding forest cover (km^2^) on the probability of successful colonization for 70 plantation landscapes.**

| **Parametric (linear) terms** | **Estimate** | **SE** | ***z* value** | ***P*** |
| --- | --- | --- | --- | --- |
| Intercept | -6.196 | 0.6004 | -10.32 | <0.0001 |
| HCVA Scenario 2 (current forest) | 0.1796 | 0.2799 | 0.642 | 0.5211 |
| HCVA Scenario 3 (full forest) | 1.007 | 0.2887 | 3.488 | <0.0001 |
| Dispersal (1 km) | 2.353 | 0.3809 | 6.176 | <0.0001 |
| Dispersal (3 km) | 5.522 | 0.475 | 11.62 | <0.0001 |
| Dispersal (5 km) | 7.348 | 0.557 | 13.19 | <0.0001 |
| Dispersal (10 km) | 10.45 | 0.910 | 11.48 | <0.0001 |
| Surrounding forest (km^2^) | 0.003 | 0.0006 | 4.989 | <0.0001 |
| **Smoothed (non-linear) terms** | **edf** | **Ref.df** | **Chi.sq** | ***P*** |
| Latitude, Longitude (interaction) | 21.75 | 25.85 | 112.8 | <0.0001 |

**Figure S4. Probabilities of successful colonization of oil palm landscapes across High Conservation Value Area (HCVA) forest cover scenarios: brown shading = no forest cover scenario, light green shading = current (2015) forest cover scenario, and dark green shading = full forest cover scenario. Probabilities are predicted values from the Generalized Additive Model (GAM; binomial logistic regression) where all covariates are held constant (i.e. at their mean values) and where dispersal abilty was excluded from the model. Bars represent standard errors.**

**
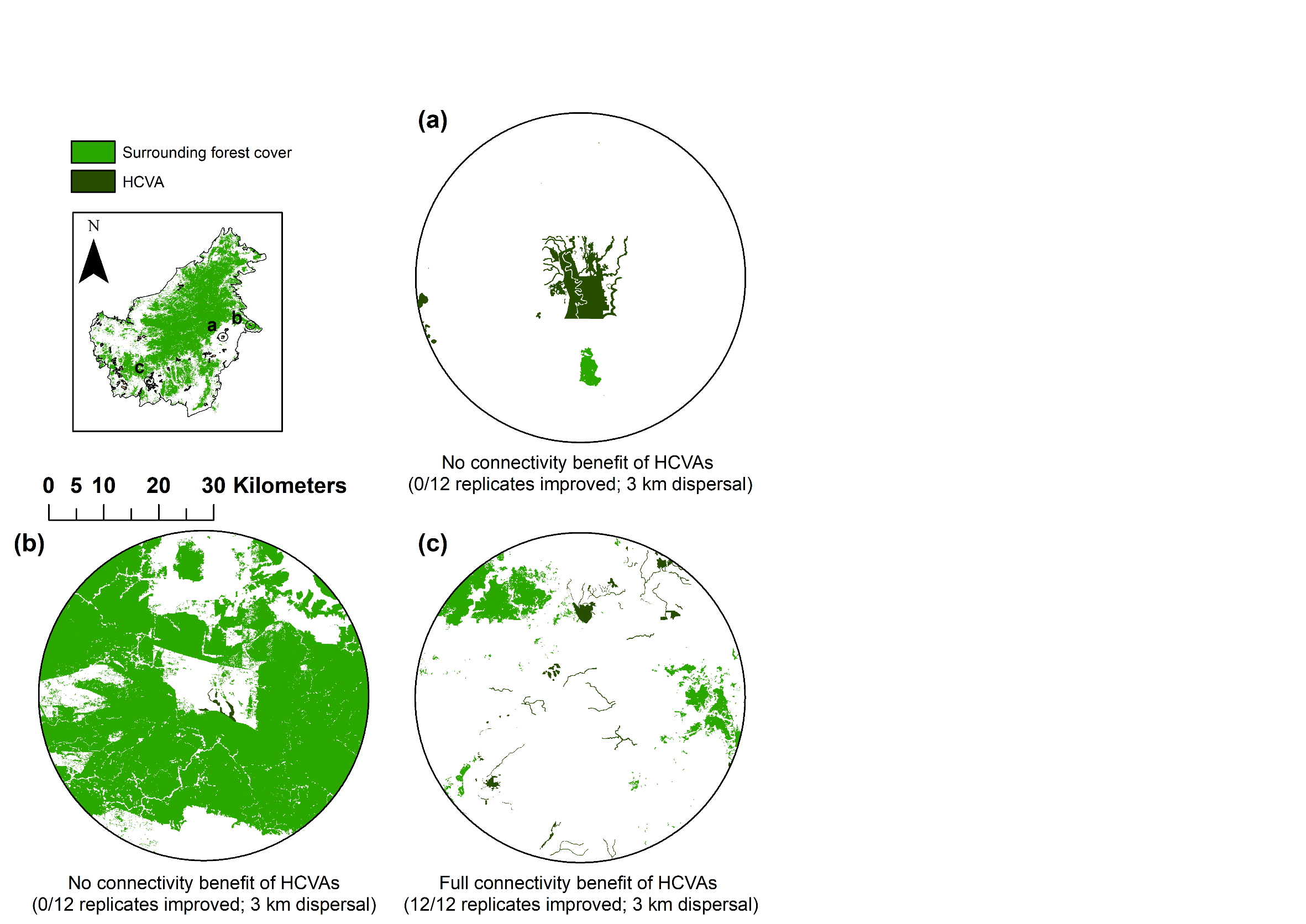
**

**Figure S5. Example plantation landscapes used to test the connectivity benefits of High Conservation Value Areas (HCVAs) using an incidence function model (IFM). Modelled landscapes are centred on New Planting Procedure (NPP) assessment plantations. Scenario (a) reflects an oil palm landscape where full forest cover in HCVAs (dark green shading) made little improvement to landscape connectivity, due to lack of forest cover surrounding the plantation (light green shading). Scenario (b) also reflects a landscape whereby full forest cover within HCVAs made little improvement to landscape connectivity, due to the large amount of surrounding forest cover and the relatively small HCVA area. Whilst scenario (c) reflects a landscape whereby full forest cover in HCVAs had large connectivity benefits for certain forest populations.**

**Appendix S3. Examining the differences in total HCVA area and forest cover between completely new developments and ongoing plantings.**

**Methods:**

To determine differences in total HCVA area (km^2^) and forest cover between completely new developments and ongoing plantings, we compared the percentage of total plantation area that was designated as HCV across completely new developments (i.e. planted following the NPP assessment; *N* = 23) and ongoing plantings (developed before 2010; *N* = 47), and compared percentage forest cover of HCVAs across these plantation types. We also calculated the percentage of the total plantation area that comprised non-HCVA forest. As data did not follow a normal distribution, we compared differences in the total HCVA area and the percentage forest cover within HCVAs in new developments and ongoing plantings using a Mann-Whitney U test. Additionally, we also used a Mann-Whitney U test to compare the percentage of total plantation area that contained non-HCVA forest between new developments and ongoing plantings.

**Results:**

The average percentage of total plantation area designated as HCVA was similar for completely new developments (12%; *N* = 23) and ongoing plantings (12%; *N* = 47) (*W*= 530; *P* = 0.90). However, HCVAs in new developments contained significantly more forest than those that were part of ongoing plantings (27% versus 18%, respectively) (*W*= 702; *P* = 0.04). New developments also contained a higher percentage of non-HCVA forest compared to ongoing plantings (14% versus 6%, respectively), but this difference was marginally insignificant (*W*= 68.2.5; *P* = 0.08).

**Appendix S4. Examining the connectivity benefits of HCVAs using least-cost models.**

**Methods:**

To determine whether the connectivity benefits of HCVAs were robust to our metric of connectivity, we calculated least-cost paths (e.g. see Adriaensen et al., 2003) across each of the 70 plantation landscapes and for the three different HCVA scenarios: assuming HCVAs were (a) absent and contained no forest cover (‘no forest’), (b) present with current (2015) forest cover (‘current forest’), or (c) present with full (100%) forest cover (‘full forest’), using the standard GRASS GIS (version 7.4) *r.cost* function. The *r.cost* function is based on a least-cost path algorithm (see GRASS Development Team, 2019 for details) and calculates the cumulative cost of moving between geographic locations (e.g. source and target grid-cells) on an input raster whose grid-cell values represent cost. We created two different resistance surface scenarios (30 m resolution raster grids) in which: (a) forest grid-cells were given a resistance (cost) value of one and non-forest (matrix) grid-cells were given a resistance value of 100 (resistance scenario 1), and (b) forest grid-cells were given a value of zero and non-forest grid-cells were given a resistance value of one (resistance scenario 2). Hence, in resistance scenario 1, the cost of traversing a non-forest grid-cell was 100 times greater than traversing a forest grid-cell. For each of the 70 plantation landscapes, we calculated least-cost paths for six directions across each landscape (i.e. directions 1-6 in Figure 2b), and recorded the overall cost of each least-cost path (i.e. the sum of resistance values of the grid-cells along the path). Resistance scenario 1 was chosen as it would likely yield a high level of variation between our 70 plantations landscapes, whilst scenario 2 was chosen as the final cost value represented the number of 30 m matrix grid-cells that must be traversed across the least-cost path between the source and target grid-cells. We then compared the overall least-cost distance values across all plantation landscapes to determine whether HCVA scenario affected the overall cost.

**Results:**

For resistance scenario 1 (where forest grid-cells were given a resistance value of one and non-forest (matrix) grid-cells were given a resistance value of 100), the cost of crossing plantation landscapes with no HCVAs (i.e. ‘no forest’ scenario) was 1.02 (92004/89861; ~2.4%) and 1.26 (92004/72856; ~26%) times more than landscapes with HCVAs that contained current forest cover (‘current forest’ scenario) and full forest cover (‘full forest’ scenario), respectively (Figure S6a). Results were similar for resistance scenario 2 (where forest grid-cells were given a value of zero and non-forest grid-cells were given a resistance value of one) (Figure S6b). Least-cost model results are comparable to the overall average results of the IFM (averaging over dispersal distances) whereby landscapes with current forest cover in HCVAs were 2.4% better connected than landscapes with no HCVAs, and landscapes with reforested HCVAs were 13.2% better connected. Note that to enable comparison, improvement values presented here were calculated from raw IFM output probabilities and are not predicted probabilities from the GAM, where all covariates were held constant (as presented in the main text). Overall, the relative improvement of reforesting HCVAs compared to landscapes with no HCVAs is slightly greater when least-cost values are the chosen metric of connectivity. Similarity of results is to be expected because both metrics are affected by the total amount of habitat in the landscape, and its spatial arrangement. For reference, across all landscapes the full forest scenario contained 3.3% (1233/37371 km^2^; area of forest in HCVAs in the full forest scenario/total landscape forest area) more forest than the no forest scenario, and the current forest scenario contained 0.72% (268/37371 km^2^; area of forest in HCVAs in the current forest scenario/total landscape forest area) more forest. The fact that relative improvements in connectivity are more substantial than relative improvements in forest cover underlines the importance of considering how landscapes function, even when limited information is available to parameterize models.

**Figure S6. Cost values produced by least-cost models run for different High Conservation Value Area (HCVA) forest cover scenarios: brown shading = no forest cover scenario, light green shading = current (2015) forest cover scenario, and dark green shading = full forest cover scenario, and for two different resistance scenarios. In (a) (resistance scenario 1) forest grid-cells were given a resistance value of one and non-forest (matrix) grid-cells were given a resistance value of 100. In (b) (resistance scenario 2) forest grid-cells were given a value of zero and non-forest grid-cells were given a resistance value of one. Bars represent standard errors.**

**(a)**

**(b)**

**Literature cited**

Adriaensen, F., Chardon, J. P., De Blust, G., Swinnen, E., Villalba, S., Gulinck, H., & Matthysen, E. (2003). The application of ‘least-cost’ modelling as a functional landscape model. *Landscape and Urban Planning*, *64*(4), 233–247. https://doi.org/10.1016/S0169-2046(02)00242-6

Bates, D., Mächler, M., Bolker, B. M., & Walker, S.C. (2015). Fitting linear mixed-effects models using lme4. *Journal of Statistical Software*, *67*(1), 1–48. https://doi.org/10.18637/jss.v067.i01

Corlett, R. T. (2009). Seed dispersal distances and plant migration potential in tropical East Asia. *Biotropica*, *41*(5), 592–598. https://doi.org/10.1111/j.1744-7429.2009.00503.x

Gaveau, D. L. A., Sheil, D., Husnayaen, , Salim, M. A., Arjasakusuma, S., Ancrenaz, M., … Meijaard, E. (2016). Rapid conversions and avoided deforestation: Examining four decades of industrial plantation expansion in Borneo. *Scientific Reports*, *6*, 32017. https://doi.org/10.1038/srep32017

GRASS Development Team. 2019. Geographic Resources Analysis Support System (GRASS)

Software, Version 7.4. Retrieved from http://grass.osgeo.org

Hanski, I. (1994). A practical model of metapopulation dynamics. *Journal of Animal Ecology*, *63*(1), 151-162. https://doi.org/10.2307/5591

Hodgson, J.A., Thomas, C. D., Cinderby, S., Cambridge, H., Evans, P., & Hill, J. K. (2011). Habitat re-creation strategies for promoting adaptation of species to climate change. *Conservation Letters*, *4*(4), 289–297. https://doi.org/10.1111/j.1755-263X.2011.00177.x

Laurance, W. F., Lovejoy, T. E., Vasconcelos, H. L., Bruna, E. M., Didham, R. K., Stouffer, P. C., … Sampaio, E. (2002). Ecosystem decay of Amazonian forest fragments: A 22-year investigation. *Conservation Biology*, *16*(3), 605–618. https://doi.org/10.1046/j.1523-1739.2002.01025.x

Lucey, J. M., Palmer, G., Yeong, K. L., Edwards, D. P., Senior, M. J. M., Scriven, S. A., … Hill, J. K. (2017). Reframing the evidence base for policy-relevance to increase impact: A case study on forest fragmentation in the oil palm sector. *Journal of Applied Ecology*, *54*(3), 731–736. https://doi.org/10.1111/1365-2664.12845

Malohlava, V., & Bocak, L. (2010). Evidence of extreme habitat stability in a Southeast Asian biodiversity hotspot based on the evolutionary analysis of neotenic net-winged beetles. *Molecular Ecology*, *19*(21), 4800–4811. https://doi.org/10.1111/j.1365-294X.2010.04850.x

Marchant, N. C., Purwanto, A., Harsanto, F. A., Boyd, N. S., Harrison, M. E., & Houlihan, P. R. (2015). ‘Random-flight’ dispersal in tropical fruit-feeding butterflies? High mobility, long lifespans and no home ranges. *Ecological Entomology*, *40*(6), 696–706. https://doi.org/10.1111/een.12239

Scriven, S. A., Hodgson, J. A., McClean, C. J., & Hill, J. K. (2015). Protected areas in Borneo may fail to conserve tropical forest biodiversity under climate change. *Biological Conservation*, *184*, 414–423. https://doi.org/10.1016/j.biocon.2015.02.018

Scroggie, M. P. & Clemann, N. (2009). Handling‐related tail loss in an endangered skink: Incidence, correlates and a possible solution. *Journal of Zoology*, *277*(3), 214-220. https://doi.org/10.1111/j.1469-7998.2008.00528.x

Wood, S. N. (2006). *Generalized additive models: An introduction with R* (1^st^ ed.; pp. 384). Boca Raton, FL: Chapman & Hall/CRC.
